# Supplementary material for: Antispasmodic Effect of Valeriana pilosa Root Essential Oil and Potential Mechanisms of Action: Ex Vivo and In Silico Studies
Source: Pharmaceutics. 2023 Aug 2;15(8):2072. doi: 10.3390/pharmaceutics15082072 (PMC10458982; doi:10.3390/pharmaceutics15082072)
Supplement: Supplementary file 1 [file pharmaceutics-15-02072-s001.zip › pharmaceutics-2527864-SI.pdf]

Antispasmodic Effect of *Valeriana pilosa* Root Essential Oil and Potential Mechanisms of Action: Ex Vivo and In Silico Studies

Roberto O. Ybañez-Julca [1,*], Ricardo Pino-Ríos [2,3], Iván M. Quispe-Díaz [1], Daniel Asunción-Alvarez [1], Edwin E. Acuña-Tarrillo [1], Elena Mantilla-Rodríguez [1], Patricia Minchan-Herrera [1], Marcelo A. Catalán [4], Liz Zevallos-Escobar [5], Edison Vásquez-Corales [5], Osvaldo Yáñez [6], Wilfredo Gutiérrez-Alvarado [7] and Julio Benites [2,3,*]

[1] Facultad de Farmacia y Bioquímica, Universidad Nacional de Trujillo, Trujillo 13011, Perú; rybanez@unitru.edu.pe(R.O.Y.-J.); iquispe@unitru.edu.pe (I.M.Q.-D.); hasuncion@unitru.edu.pe (D.A.-A.); eacunat@unitru.edu.pe (E.E.A-T.); jgonzalesn@unitru.edu.pe (D.G.-N.); amnatilla@unitru.edu.pe (E.M-R); pminchan@unitru.edu.pe (P.M.-H.)

[2] Química y Farmacia, Facultad de Ciencias de la Salud, Universidad Arturo Prat, Casilla 121, Iquique 1100000, Chile; juliob@unap.cl (J.B.)

[3] Instituto de Estudios de la Salud, Universidad Arturo Prat, Casilla 121, Iquique 1100000, Chile; rpinorios@unap.cl (R.P.-R.)

[4] Instituto de Fisiología, Facultad de Medicina, Universidad Austral de Chile, Valdivia 5090000, Chile; marcelo.catalan@uach.cl (M.A.C.)

[5] Escuela de Farmacia y Bioquímica, Universidad Católica Los Ángeles de Chimbote, Chimbote 02801, Perú; lzevallose@uladech.edu.pe (L.Z.-E.); evasquezc@uladech.edu.pe (E.V.-C.)

[6] Facultad de Ingeniería y Negocios, Universidad de las Américas, Santiago 7500000, Chile; oyanez@udla.cl (O.Y.)

[7] Facultad de Farmacia y Bioquímica, Universidad Nacional de la Amazonía Peruana, Iquitos 16001, Perú; Wilfredo.gutierrez@unapiquitos@edu.pe (W.G.-A)

* Correspondence: rybanez@unitru.edu.pe (R.O.Y.-J.); juliob@unap.cl (J.B.); Tel.: +51-0449-7634-5993 (R.O.Y.-J.); Tel.: +56-57-2252-6275 (J.B.)

SUPPORTING INFORMATION

Table S1. Smiles for all compounds in *Valeriana pilosa* essential oil.

N°	Compounds	Smiles
1	Isovaleric acid	CC(CC(=O)O)C
2	Tricyclene	C[C@]12[C@@H]3[C@H]1C[C@H](C2(C)C)C3
3	α-Thujene	CC1=CC[C@@]2([C@H]1C2)C(C)C
4	α-Pinene	CC1=CC[C@H]2C[C@@H]1C2(C)C
5	Camphene	C=C1[C@H]2CC[C@@H](C1(C)C)C2
6	3-Methyl valeric acid	C[C@H](CC)CC(=O)O
7	Sabinene	C=C1CC[C@@]2([C@H]1C2)C(C)C
8	1-Octen-3-ol	CCCCC[C@H](C=C)O
9	β-Pinene	C=C1CC[C@H]2C[C@@H]1C2(C)C
10	Myrcene	C=CC(=C)CCC=C(C)C
11	Limonene	CC1=CC[C@@H](CC1)C(=C)C
12	p-Cymene	Cc1ccc(cc1)C(C)C
13	1,8-Cineole	C[C@]12CC[C@H](CC1)C(O2)(C)C
14	Linalool	C=C[C@](CCC=C(C)C)(O)C
15	Isopentyl isovalerate	CC(CCOC(=O)CC(C)C)C
16	Camphor	O=C1C[C@H]2C([C@]1(C)CC2)(C)C
17	Menthone	C[C@@H]1CC[C@@H](C(=O)C1)C(C)C
18	Isomenthone	C[C@@H]1CC[C@@H](C(=O)C1)C(C)C
19	Borneol	O[C@@H]1C[C@H]2C([C@]1(C)CC2)(C)C
20	Neomenthol	C[C@H]1CC[C@H]([C@@H](C1)O)C(C)C
21	Menthol	C[C@@H]1CC[C@H]([C@H](C1)O)C(C)C
22	Carvone	CC(=C)[C@@H]1CC=C(C(=O)C1)C
23	Menthyl acetate	C[C@@H]1CC[C@H]([C@@H](C1)OC(=O)C)C(C)C
24	α-Cubebene	CC([C@H]1CC[C@H]([C@@]23[C@@H]1[C@@H]2C(=CC3)C)C)C
25	Cyclosativene	CC([C@H]1CC[C@@H]([C@@]23[C@@H]1[C@@H]1[C@@H]([C@]21C)C3)C)C
26	α-Copaene	C[C@@H]1CC[C@@]2([C@@H]3[C@H]1[C@H]2C(=CC3)C)C
27	β-Patchoulene	C[C@@H]1CCC2=C1C[C@H]1CC[C@@]2(C1(C)C)C
28	β-Bourbonene	CC([C@@H]1CC[C@@]2([C@H]1[C@@H]1C(=C)CC[C@H]21)C)C
29	β-Elemene	C=C[C@@]1(C)CC[C@H](C[C@H]1C(=C)C)C(=C)C
30	β-Caryophyllene	C[C@H]1CCCC(=C)[C@H]2[C@H](CC1)C(C2)(C)C
31	Seychellene	C[C@H]1CC[C@@]2([C@@]3([C@H]1C[C@H](C2=C)CC3)C)C
32	α-Guaiene	CC(=C)[C@@H]1CC[C@@H](C2=C(C1)[C@@H](C)CC2)C
33	α-Humulene	CC1=CCC(C)(C)C=CCC(=CCC1)C
34	allo-Aromadendrene	C[C@H]1CC[C@@H]2[C@H]1[C@@H]1[C@@H](C1(C)C)CCC2=C
35	α-Patchoulene	C[C@H]1CC[C@]23[C@@H]1C[C@@H](C3(C)C)CC=C2C

36	γ-Muurolene	CC1=C[C@H]2[C@@H](CC1)C(=C)CC[C@@H]2C(C)C
37	Germacrene-D	CC1=CCCC(=C)C=C[C@@H](CC1)C(C)C
38	Valencene	CC(=C)[C@@H]1CCC2=CCC[C@H]([C@]2(C1)C)C
39	Eremophyllene	CC(=C)[C@@H]1CC[C@@H]2[C@](C1)(C)C(=CCC2)C
40	γ-Cadinene	CC1=C[C@@H]2[C@H](CC1)C(=C)CC[C@@H]2C(C)C
41	7-epi-α-Selinene	CC1=CCC[C@]2([C@@H]1C[C@@H](CC2)C(=C)C)C
42	δ-Cadinene	CC1=C[C@H]2C(=C(C)CC[C@H]2C(C)C)CC1
43	Spathulenol	C=C1CC[C@H]2[C@@H]([C@@H]3[C@@H]1CC[C@]3(C)O)C2(C)C
44	β-Caryophyllene oxide	C=C1CC[C@@H]2O[C@@]2(CC[C@@H]2[C@@H]1CC2(C)C)C
45	T-Cadinol	CC1=C[C@H]2[C@@H](CC1)[C@@](C)(O)CC[C@H]2C(C)C
46	δ-Cadinol	CC1=C[C@H]2[C@@H](CC1)[C@@](C)(O)CC[C@H]2C(C)C
47	Patchoulol	C[C@H]1CC[C@@]2([C@@]3([C@H]1C[C@H](C2(C)C)CC3)C)O

Table S2. Composition (in %) of *Valeriana pilosa* essential oil. Taken from reference 20.

N°	Components	Composition (in %)
1	Isovaleric acid	2.6
2	Tricyclene	0
3	α-Thujene	0.1
4	α-Pinene	3.7
5	Camphene	1.4
6	3-Methyl valeric acid	3.1
7	Sabinene	0.4
8	1-Octen-3-ol	0
9	β-Pinene	0.6
10	Myrcene	0.1
11	Limonene	3.2
12	p-Cymene	0
13	1,8-Cineole	4.3
14	Linalool	0.1
15	Isopentyl isovalerate	0
16	Camphor	0.2
17	Menthone	0.8
18	Isomenthone	0.2
19	Borneol	0
20	Neomenthol	0
21	Menthol	1.2
22	Carvone	0.1
23	Menthyl acetate	1.4
24	α-Cubebene	0.2
25	Cyclosativene	0.1
26	α-Copaene	1
27	β-Patchoulene	0.4
28	β-Bourbonene	0.4
29	β-Elemene	0.8
30	β-Caryophyllene	3.5
31	Seychellene	7.6
32	α-Guaiene	4.1
33	α-Humulene	6.1
34	allo-Aromadendrene	2.2
35	α-Patchoulene	5.8
36	γ-Muurolene	1
37	Germacrene-D	0.4

38	Valencene	0.3
39	Eremophyllene	0.3
40	γ-Cadinene	0.2
41	7-epi-α-Selinene	2.5
42	δ-Cadinene	0.8
43	Spathulenol	1.6
44	β-Caryophyllene oxide	2.9
45	T-Cadinol	0.5
46	δ-Cadinol	0.5
47	Patchoulol	20.8

Table S3. $\Delta E_{binding}$ (kcal·mol^{-1}), Kd, *LE* (kcal·mol^{-1}), *BEI*(kDa), *LLE*, $IE_{norm.binding}$ (kcal·mol^{-1}) obtained after molecular docking between *Valeriana pilosa* essential oil compounds and M_3 Muscarinic Acetylcholine Receptor (PDBID: 4DAJ).

N°	Compounds	$\Delta E_{binding}$ (kcal·mol^{-1})	K_d	*LE* (kcal·mol^{-1})	*BEI*(kDa)	*LLE*	$IE_{norm.binding}$ (kcal·mol^{-1})
1	Isovaleric acid	-4.5	5.04E-04	0.6	32.3	2.2	-1.7
2	Tricyclene	-6.5	1.72E-05	0.7	35.0	2.1	-2.1
3	α-Thujene	-6.7	1.23E-05	0.7	36.0	1.9	-2.1
4	α-Pinene	-6.8	1.04E-05	0.7	36.6	2.0	-2.2
5	Camphene	-6.7	1.23E-05	0.7	36.0	1.9	-2.1
6	3-Methyl valeric acid	-4.8	3.04E-04	0.6	30.3	2.0	-1.7
7	Sabinene	-6.8	1.04E-05	0.7	36.6	2.0	-2.2
8	1-Octen-3-ol	-5.1	1.83E-04	0.6	29.2	1.6	-1.7
9	β-Pinene	-6.8	1.04E-05	0.7	36.6	2.0	-2.2
10	Myrcene	-5.9	4.74E-05	0.6	31.7	0.8	-1.9
11	Limonene	-6.5	1.72E-05	0.7	35.0	1.5	-2.1
12	p-Cymene	-6.5	1.72E-05	0.7	35.5	1.6	-2.1
13	1,8-Cineole	-6.7	1.23E-05	0.6	31.8	2.2	-2.0
14	Linalool	-5.9	4.74E-05	0.5	28.0	1.7	-1.8
15	Isopentyl isovalerate	-6.0	4.01E-05	0.5	25.5	1.8	-1.7
16	Camphor	-6.7	1.23E-05	0.6	32.3	2.5	-2.0
17	Menthone	-6.7	1.23E-05	0.6	31.8	2.3	-2.0
18	Isomenthone	-6.7	1.23E-05	0.6	31.8	2.3	-2.0
19	Borneol	-6.5	1.72E-05	0.6	30.9	2.6	-2.0
20	Neomenthol	-6.6	1.46E-05	0.6	31.0	2.4	-2.0
21	Menthol	-6.3	2.41E-05	0.6	29.5	2.2	-1.9
22	Carvone	-6.9	8.77E-06	0.6	33.7	2.6	-2.1
23	Menthyl acetate	-7.4	3.77E-06	0.5	27.3	2.4	-2.0
24	α-Cubebene	-9.0	2.54E-07	0.6	32.3	2.3	-2.3
25	Cyclosativene	-8.9	3.00E-07	0.6	31.9	2.6	-2.3
26	α-Copaene	-8.2	9.78E-07	0.6	34.1	2.4	-2.3
27	β-Patchoulene	-9.4	1.29E-07	0.6	33.7	2.3	-2.4
28	β-Bourbonene	-9.1	2.14E-07	0.6	32.6	2.4	-2.3
29	β-Elemene	-8.0	1.37E-06	0.5	28.7	1.1	-2.1
30	β-Caryophyllene	-9.1	2.14E-07	0.6	32.3	1.9	-2.3
31	Seychellene	-8.4	6.98E-07	0.6	30.1	1.7	-2.2
32	α-Guaiene	-9.0	2.54E-07	0.6	32.3	1.9	-2.3
33	α-Humulene	-9.1	2.14E-07	0.6	32.6	1.6	-2.3

34	allo-Aromadendrene	-8.6	4.98E-07	0.6	30.8	2.0	-2.2
35	α-Patchoulene	-8.7	4.21E-07	0.6	31.2	2.0	-2.2
36	γ-Muurolene	-9.2	1.81E-07	0.6	33.0	2.2	-2.4
37	Germacrene-D	-8.8	3.55E-07	0.6	31.6	1.6	-2.3
38	Valencene	-9.0	2.54E-07	0.6	32.3	1.9	-2.3
39	Eremophyllene	-8.9	3.00E-07	0.6	31.9	1.8	-2.3
40	γ-Cadinene	-8.6	4.98E-07	0.6	30.8	1.7	-2.2
41	7-epi-α-Selinene	-8.8	3.55E-07	0.6	31.6	1.7	-2.3
42	δ-Cadinene	-9.0	2.54E-07	0.6	32.3	1.9	-2.3
43	Spathulenol	-8.6	4.98E-07	0.5	28.6	2.9	-2.2
44	β-Caryophyllene oxide	-8.3	8.26E-07	0.5	27.6	2.1	-2.1
45	T-Cadinol	-8.8	3.55E-07	0.6	29.0	2.7	-2.2
46	δ-Cadinol	-8.8	3.55E-07	0.6	29.0	2.7	-2.2
47	Patchoulol	-8.6	4.98E-07	0.5	28.3	2.7	-2.2

Table S4. $\Delta E_{binding}$ (kcal·mol⁻¹), Kd, *LE* (kcal·mol⁻¹), *BEI*(kDa), *LLE*, $IE_{norm.binding}$ (kcal·mol⁻¹) obtained after molecular docking between *Valeriana pilosa* essential oil compounds and M_2 Muscarinic Acetylcholine Receptor (PDBID: 3UON).

N°	Compounds	$\Delta E_{binding}$ (kcal·mol⁻¹)	K_d	*LE* (kcal·mol⁻¹)	*BEI*(kDa)	*LLE*	$IE_{norm.binding}$ (kcal·mol⁻¹)
1	Isovaleric acid	-4.8	8.35E-04	0.6	30.1	2.0	-1.6
2	Tricyclene	-6.8	5.96E-04	0.4	23.7	0.5	-1.4
3	α-Thujene	-6.5	4.25E-04	0.5	24.7	0.4	-1.5
4	α-Pinene	-6.5	7.06E-04	0.4	23.1	0.2	-1.4
5	Camphene	-6.9	4.25E-04	0.5	24.7	0.4	-1.5
6	3-Methyl valeric acid	-4.7	5.96E-04	0.6	27.8	1.7	-1.6
7	Sabinene	-6.5	3.59E-04	0.5	25.3	0.4	-1.5
8	1-Octen-3-ol	-5.2	1.94E-03	0.4	21.1	0.6	-1.2
9	β-Pinene	-6.6	5.96E-04	0.4	23.7	0.2	-1.4
10	Myrcene	-6.0	1.94E-03	0.4	19.9	-0.8	-1.2
11	Limonene	-6.5	5.04E-04	0.5	24.2	0.0	-1.4
12	p-Cymene	-6.6	4.25E-04	0.5	25.1	0.3	-1.5
13	1,8-Cineole	-6.7	5.96E-04	0.4	20.9	0.5	-1.3
14	Linalool	-6.0	5.04E-04	0.4	21.4	0.6	-1.4
15	Isopentyl isovalerate	-5.8	7.06E-04	0.4	18.3	0.5	-1.2
16	Camphor	-6.9	8.35E-04	0.4	20.2	0.7	-1.3
17	Menthone	-6.9	5.96E-04	0.4	20.9	0.6	-1.3
18	Isomenthone	-6.9	5.96E-04	0.4	20.9	0.6	-1.3
19	Borneol	-6.8	9.89E-04	0.4	19.5	0.8	-1.2
20	Neomenthol	-6.8	8.35E-04	0.4	19.7	0.6	-1.3
21	Menthol	-6.4	8.35E-04	0.4	19.7	0.6	-1.3
22	Carvone	-6.9	3.59E-04	0.4	22.9	1.0	-1.4
23	Menthyl acetate	-7.5	3.59E-04	0.3	17.4	0.4	-1.3
24	α-Cubebene	-8.5	9.32E-05	0.4	19.7	-0.2	-1.4
25	Cyclosativene	-8.5	1.10E-04	0.4	19.4	0.0	-1.4
26	α-Copaene	-8.2	1.31E-04	0.4	22.0	0.2	-1.5
27	β-Patchoulene	-9.1	5.61E-05	0.4	20.8	-0.3	-1.5
28	β-Bourbonene	-8.9	1.83E-04	0.3	18.3	-0.5	-1.3
29	β-Elemene	-8.1	2.17E-04	0.3	17.9	-1.1	-1.3
30	β-Caryophyllene	-9.0	7.87E-05	0.4	19.9	-0.7	-1.4
31	Seychellene	-7.9	1.10E-04	0.4	19.4	-0.5	-1.4
32	α-Guaiene	-8.6	6.65E-05	0.4	20.4	-0.5	-1.5
33	α-Humulene	-8.6	1.31E-04	0.4	19.0	-1.2	-1.4

34	allo-Aromadendrene	-8.3	1.10E-04	0.4	19.4	-0.3	-1.4
35	α-Patchoulene	-8.3	9.32E-05	0.4	19.7	-0.4	-1.4
36	γ-Muurolene	-9.1	9.32E-05	0.4	19.7	-0.6	-1.4
37	Germacrene-D	-8.6	1.55E-04	0.3	18.6	-1.1	-1.3
38	Valencene	-8.5	7.87E-05	0.4	20.1	-0.6	-1.4
39	Eremophyllene	-9.1	9.32E-05	0.4	19.7	-0.7	-1.4
40	γ-Cadinene	-8.6	9.32E-05	0.4	19.7	-0.6	-1.4
41	7-epi-α-Selinene	-8.3	1.10E-04	0.4	19.4	-0.8	-1.4
42	δ-Cadinene	-8.8	1.10E-04	0.4	19.4	-0.8	-1.4
43	Spathulenol	-8.6	1.10E-04	0.3	18.0	0.6	-1.4
44	β-Caryophyllene oxide	-8.1	9.32E-05	0.3	18.3	0.1	-1.4
45	T-Cadinol	-8.5	1.10E-04	0.3	17.8	0.2	-1.4
46	δ-Cadinol	-8.4	1.10E-04	0.3	17.8	0.2	-1.4
47	Patchoulol	-7.8	1.55E-04	0.3	17.1	0.2	-1.3

Table S5. $\Delta E_{binding}$ (kcal·mol^{-1}), Kd, *LE* (kcal·mol^{-1}), *BEI*(kDa), *LLE*, *IE* $_{norm.binding}$ (kcal·mol^{-1}) obtained after molecular docking between *Valeriana pilosa* essential oil compounds and L-type Volgate-gated calcium channel (PDBID: 5V2P).

N°	Compounds	$\Delta E_{binding}$ (kcal·mol^{-1})	K_d	*LE* (kcal·mol^{-1})	*BEI*(kDa)	*LLE*	$IE_{norm.binding}$ (kcal·mol^{-1})
1	Isovaleric acid	-4.2	8.35E-04	0.6	30.1	2.0	-1.6
2	Tricyclene	-4.4	5.96E-04	0.4	23.7	0.5	-1.4
3	α-Thujene	-4.6	4.25E-04	0.5	24.7	0.4	-1.5
4	α-Pinene	-4.3	7.06E-04	0.4	23.1	0.2	-1.4
5	Camphene	-4.6	4.25E-04	0.5	24.7	0.4	-1.5
6	3-Methyl valeric acid	-4.4	5.96E-04	0.6	27.8	1.7	-1.6
7	Sabinene	-4.7	3.59E-04	0.5	25.3	0.4	-1.5
8	1-Octen-3-ol	-3.7	1.94E-03	0.4	21.1	0.6	-1.2
9	β-Pinene	-4.4	5.96E-04	0.4	23.7	0.2	-1.4
10	Myrcene	-3.7	1.94E-03	0.4	19.9	-0.8	-1.2
11	Limonene	-4.5	5.04E-04	0.5	24.2	0.0	-1.4
12	p-Cymene	-4.6	4.25E-04	0.5	25.1	0.3	-1.5
13	1,8-Cineole	-4.4	5.96E-04	0.4	20.9	0.5	-1.3
14	Linalool	-4.5	5.04E-04	0.4	21.4	0.6	-1.4
15	Isopentyl isovalerate	-4.3	7.06E-04	0.4	18.3	0.5	-1.2
16	Camphor	-4.2	8.35E-04	0.4	20.2	0.7	-1.3
17	Menthone	-4.4	5.96E-04	0.4	20.9	0.6	-1.3
18	Isomenthone	-4.4	5.96E-04	0.4	20.9	0.6	-1.3
19	Borneol	-4.1	9.89E-04	0.4	19.5	0.8	-1.2
20	Neomenthol	-4.2	8.35E-04	0.4	19.7	0.6	-1.3
21	Menthol	-4.2	8.35E-04	0.4	19.7	0.6	-1.3
22	Carvone	-4.7	3.59E-04	0.4	22.9	1.0	-1.4
23	Menthyl acetate	-4.7	3.59E-04	0.3	17.4	0.4	-1.3
24	α-Cubebene	-5.5	9.32E-05	0.4	19.7	-0.2	-1.4
25	Cyclosativene	-5.4	1.10E-04	0.4	19.4	0.0	-1.4
26	α-Copaene	-5.3	1.31E-04	0.4	22.0	0.2	-1.5
27	β-Patchoulene	-5.8	5.61E-05	0.4	20.8	-0.3	-1.5
28	β-Bourbonene	-5.1	1.83E-04	0.3	18.3	-0.5	-1.3
29	β-Elemene	-5.0	2.17E-04	0.3	17.9	-1.1	-1.3
30	β-Caryophyllene	-5.6	7.87E-05	0.4	19.9	-0.7	-1.4
31	Seychellene	-5.4	1.10E-04	0.4	19.4	-0.5	-1.4
32	α-Guaiene	-5.7	6.65E-05	0.4	20.4	-0.5	-1.5
33	α-Humulene	-5.3	1.31E-04	0.4	19.0	-1.2	-1.4

34	allo-Aromadendrene	-5.4	1.10E-04	0.4	19.4	-0.3	-1.4
35	α-Patchoulene	-5.5	9.32E-05	0.4	19.7	-0.4	-1.4
36	γ-Muurolene	-5.5	9.32E-05	0.4	19.7	-0.6	-1.4
37	Germacrene-D	-5.2	1.55E-04	0.3	18.6	-1.1	-1.3
38	Valencene	-5.6	7.87E-05	0.4	20.1	-0.6	-1.4
39	Eremophyllene	-5.5	9.32E-05	0.4	19.7	-0.7	-1.4
40	γ-Cadinene	-5.5	9.32E-05	0.4	19.7	-0.6	-1.4
41	7-epi-α-Selinene	-5.4	1.10E-04	0.4	19.4	-0.8	-1.4
42	δ-Cadinene	-5.4	1.10E-04	0.4	19.4	-0.8	-1.4
43	Spathulenol	-5.4	1.10E-04	0.3	18.0	0.6	-1.4
44	β-Caryophyllene oxide	-5.5	9.32E-05	0.3	18.3	0.1	-1.4
45	T-Cadinol	-5.4	1.10E-04	0.3	17.8	0.2	-1.4
46	δ-Cadinol	-5.4	1.10E-04	0.3	17.8	0.2	-1.4
47	Patchoulol	-5.2	1.55E-04	0.3	17.1	0.2	-1.3
